# Aβ1-16 controls synaptic vesicle pools at excitatory synapses via cholinergic modulation of synapsin phosphorylation

**DOI:** 10.1007/s00018-021-03835-5

**Published:** 2021-04-17

**Authors:** Daniela Anni, Eva-Maria Weiss, Debarpan Guhathakurta, Yagiz Enes Akdas, Julia Klueva, Stefanie Zeitler, Maria Andres-Alonso, Tobias Huth, Anna Fejtova

**Affiliations:** 1 Department of Psychiatry and Psychotherapy, University Hospital, Friedrich-Alexander Universität Erlangen-Nürnberg, Erlangen, Germany; 2 RG Presynaptic Plasticity, Leibniz Institute for Neurobiology, Magdeburg, Germany; 3 Institute of Physiology and Pathophysiology, Friedrich-Alexander Universität Erlangen-Nürnberg, Erlangen, Germany

**Keywords:** Amyloid beta, Alpha7 nicotinic acetylcholine receptor, Synaptic vesicle dynamics, Synapsin 1

## Abstract

Amyloid beta (Aβ) is linked to the pathology of Alzheimer’s disease (AD). At physiological concentrations, Aβ was proposed to enhance neuroplasticity and memory formation by increasing the neurotransmitter release from presynapse. However, the exact mechanisms underlying this presynaptic effect as well as specific contribution of endogenously occurring Aβ isoforms remain unclear. Here, we demonstrate that Aβ1-42 and Aβ1-16, but not Aβ17-42, increased size of the recycling pool of synaptic vesicles (SV). This presynaptic effect was driven by enhancement of endogenous cholinergic signalling via α7 nicotinic acetylcholine receptors, which led to activation of calcineurin, dephosphorylation of synapsin 1 and consequently resulted in reorganization of functional pools of SV increasing their availability for sustained neurotransmission. Our results identify synapsin 1 as a molecular target of Aβ and reveal an effect of physiological concentrations of Aβ on cholinergic modulation of glutamatergic neurotransmission. These findings provide new mechanistic insights in cholinergic dysfunction observed in AD.

**Supplementary Information:**

The online version contains supplementary material available at 10.1007/s00018-021-03835-5.

## Introduction

Amyloid beta peptide (Aβ) is tightly linked to the pathology of Alzheimer’s disease (AD). It forms amyloid plaques, which are an important neuropathological hallmark of AD [32]. Aβ arises from proteolytic cleavage of the transmembrane amyloid precursor protein (APP). High mass Aβ species (Aβ1-40 and Aβ1-42) arise from the amyloidogenic proteolytic processing of APP by a combined action of β- and γ-secretases [15, 43]. In the non-amyloidogenic pathway, the cleavage of APP by α-secretase and γ-secretase precludes the generation of high mass Aβ resulting in the liberation of the soluble APP ectodomain (sAPPα) and P3 peptides (Aβ17-40, Aβ17-42) [16]. An alternative pathway, which involves the concerted action of α- and β-secretases, was identified as source of lower mass peptides covering the N-terminal portion of Aβ [36]. The most abundant species found in the cerebrospinal fluid (CSF) are the high mass Aβ1-40 and lower mass species containing its N-terminal portion ending around amino acid (aa) 16 [35, 37, 46]. Up to date, most studies in AD focused on Aβ1-42, which is relatively low abundant in CSF, but highly prone to aggregation and therefore the seeding component of amyloid senile plaques [20]. In contrast to that, the function of the highly abundant low mass Aβ fragments and nontoxic Aβ 1–40 remains less explored.

Over the past decade, it became clear that APP and Aβ possesses central physiological functions in brains. This was supported by the observation that blockade of endogenous Aβ using specific antibodies as well ablations of APP expression impaired long-term potentiation (LTP) and memory [18, 33, 38]. Thus, the question about possible roles of Aβ in the regulation of synaptic physiology emerged as a key for understanding the mechanism of AD pathogenesis. Aβ peptides are secreted by neurons in response to neuronal activity and reach concentrations of 200 picomolar (pM) in rodent and human brains [3, 7, 23]. Acute application of 200–300 pM Aβ1-42 increased hippocampal LTP and memory [39], which involves a potentiation of spontaneous and evoked neurotransmitter (NT) release from presynapse [14]. Moreover, an increase of endogenous Aβ concentrations by interference with normal clearance or an application of pM amounts of synthetic Aβ1-40 or Aβ1-42 enhanced NT release and recycling of synaptic vesicles (SVs) in cultured neurons [1, 28]. A contribution of α7 nicotinic acetylcholine receptors (α7nAChRs) was proposed, but remains controversial. Pharmacological interference and genetic ablation of this receptor precluded the effect of Aβ1-42 on LTP and hippocampal-dependent memory [14, 39]. However, the studies addressing their contribution to the modulation of SV recycling by Aβ came to contradictory conclusions [10, 28]. Moreover, most recently effects of the low mass N-terminal Aβ fragments and/or the secreted APPα fragments containing the N-terminal Aβ portion on neuroplasticity and cognition were described, which were linked to α7nAChRs and proposed its presynaptic site of action [18, 27, 40]. These data indicate that the physiological function of the full length Aβ may reside in its N-terminal portion, however, a systematic comparison of these Aβ-derived fragments at the presynaptic level has not been performed yet.

Thus, in this study, we dissected effects of Aβ1-42 and its N- and C-terminal fragments on SV recycling. We show that the N-terminal fragment, Aβ1-16, but not the C-terminal Aβ17-42 holds all regulatory effects of Aβ1-42. Using pharmacological and genetic approaches, we confirm a key role of α7nAChRs in this process and postulate that Aβ1-16 acts as a positive allosteric modulator of these receptors. Moreover, we identified the activation of phosphatase calcineurin and consequent dephosphorylation of synapsin 1 (Syn1) as a molecular mechanism underlying the increased SVs mobilization and availability upon Aβ1-16 application. Thus, we propose that physiologically occurring Aβ1-16 enhances cholinergic modulation of glutamatergic presynapse.

## Results

### Aβ1-16 increases recycling of SVs at glutamatergic synapses

**Fig. 1:**
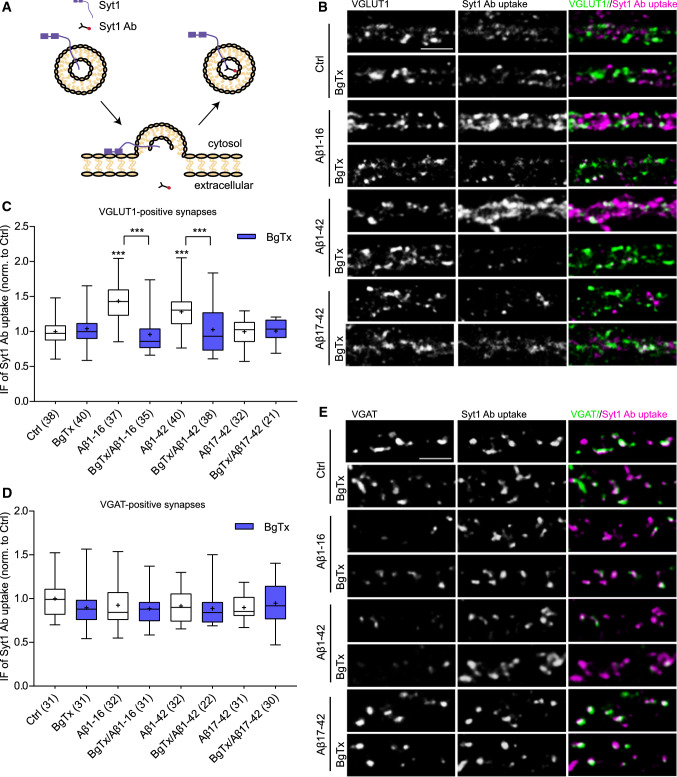
The N-terminal domain of Aβ increases the TRP of SVs via α7nAChRs at excitatory but not inhibitory synapses. **a** Schematic representation of labelling of SV recycling using Syt1 Ab. Upon depolarization, releasable SVs fuse to the plasma membrane. The lumenal domain of integral SV protein Syt1 becomes exposed to the media and available for Syt1 Ab binding. Compensatory endocytosis drives the uptake of Syt1 Ab inside of retrieved SVs. **b**, **e** Representative images of depolarization-induced Syt1 Ab uptake (magenta) in primary cortical neurons (20–21 DIV) treated with vehicle (Ctrl), Aβ1-16, Aβ1-42, or Aβ17-42 (200 pM, 1 h) in the presence or absence of BgTx (50 nM, 90 min). Antibody against VGLUT1 and VGAT (green) was used to mark excitatory and inhibitory synapses, respectively. Scale bar is 5 μm. **c**, **d** Quantifications of Syt1 Ab uptake from (B,E). Values in brackets show the number of analysed cells obtained from two to three independent preparations. In the plots, boxes indicate the interquartile range and median, whiskers minimum and maximum values, and + depicts the mean. One-way ANOVA with Dunnett´s post hoc test was used to evaluate statistical significance. Significance of comparison to control are shown about the boxes, the BgTx effects are shown above the brackets; ***p < 0.001

We have shown previously that modulation of extracellular levels of endogenous Aβ affects recycling of SVs [28]. To gain further insights into the mechanisms behind the regulation of NT release, we monitored SV recycling in living rat cortical neurons. To this end, we used a fluorescently labelled anti-synaptotagmin 1 antibody (Syt1 Ab), which was directed against the lumenal domain of this SV protein. Once added to the media, this antibody internalizes and labels SVs during their fusion with the plasma membrane (Fig. 1a), and thus the amount of SVs that undertook exo-/endocytic cycles can be approximated by the uptaken immunofluorescence (IF) at individual synapses [25, 28]. To label the total recycling pool of SVs (TRP), we induced exocytosis of all releasable vesicles by a brief (4 min) application of depolarizing media. We quantified the TRP in neurons pre-treated with vehicle or with solution containing synthetic Aβ1-16, Aβ1-42, or Aβ17-42 peptides for 1 h. First, we assessed individual excitatory synapses that were labelled post hoc using an antibody against the vesicular glutamate transporter-1 (VGLUT1). The observed Syt1 Ab uptake was significantly increased in cells treated with 200 pM Aβ1-16 and Aβ1-42 compared to vehicle-treated cells (Fig. 1b,c; Aβ1-16: 1.43 ± 0.05; Aβ1-42: 1.28 ± 0.04; Ctrl: 1 ± 0.03). To evaluate a possible requirement of α7nAChRs, 50 nM of a selective α7nAChR antagonist α-Bungarotoxin (BgTx) was applied 30 min before and during Aβ treatments. While BgTx alone had no effect on Syt1 Ab uptake in non-treated cells (Fig. 1b,c; BgTx: 1.04 ± 0.04), it interfered with the effect of both Aβ1-16 and Aβ1-42 (Fig. 1b,c; BgTx/Aβ1-16: 0.96 ± 0.04; BgTx/Aβ1-42: 1.03 ± 0.05). Interestingly, application of Aβ17-42 had no effect in presence or in absence of BgTx (Fig. 1b,c; Aβ17-42: 0.99 ± 0.03; BgTx/Aβ17-42: 1 ± 0.03). Next, we assessed TRP in GABAergic synapses that were identified using an antibody against the vesicular GABA transporter (VGAT). None of Aβ-derived peptides affected GABAergic SV recycling (Fig. 1d,e; Ctrl: 1 ± 0.04; BgTx: 0.90 ± 0.03; Aβ1-16: 0.92 ± 0.04; BgTx/Aβ1-16: 0.88 ± 0.03; Aβ1-42: 0.92 ± 0.03; BgTx/Aβ1-42: 0.89 ± 0.04; Aβ17-42: 0.90 ± 0.03; BgTx/Aβ17-42: 0.95 ± 0.04). Thus, these data constitute the N-terminal amino acids (aa) 1–16 of Aβ1-42 as the active epitope sufficient and necessary to regulate presynaptic recycling. Moreover, they indicate a role for α7nAChR in Aβ-mediated effect on TRP at excitatory, but not at inhibitory synapses.

**Fig. 2:**
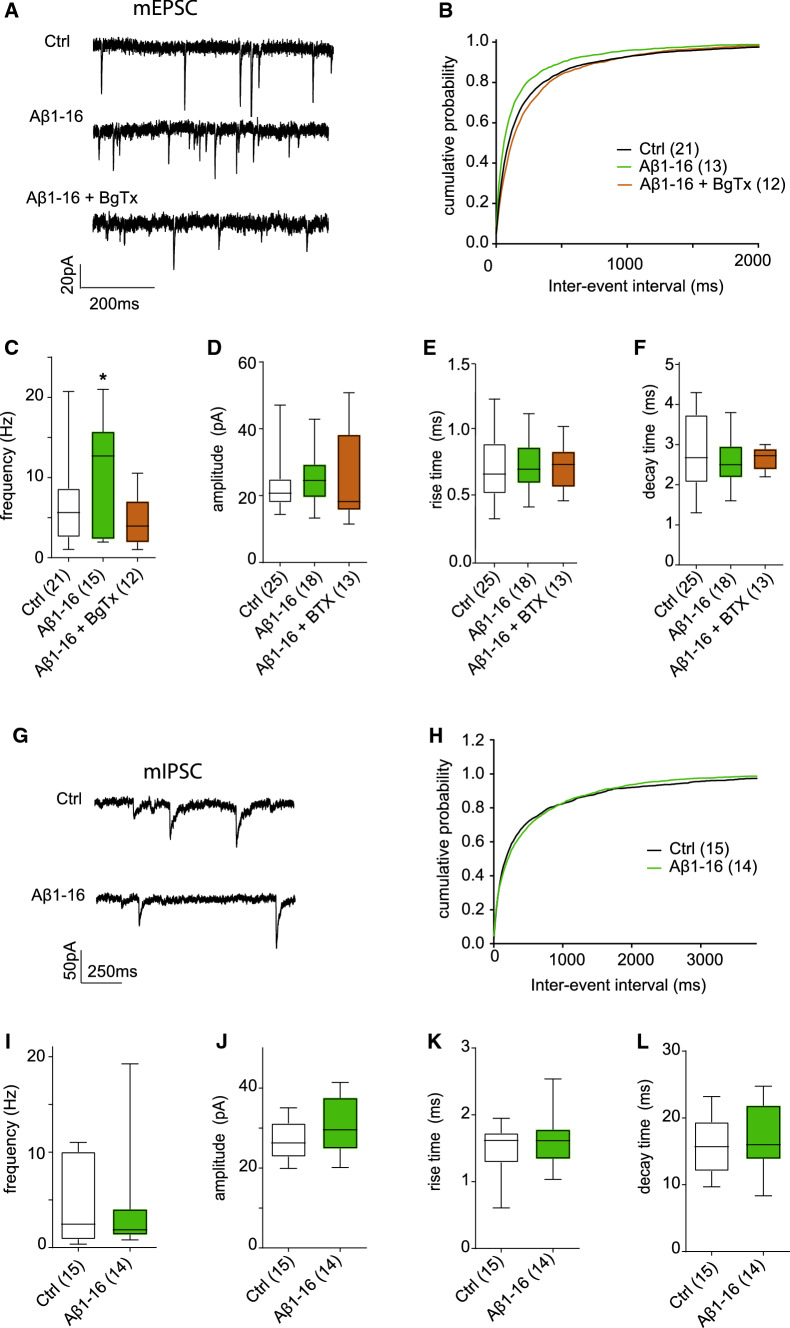
Effect of Aβ1-16 on mEPSCs and mIPSC in hippocampal neurons. **a** Examples of mEPSC traces recorded in neurons under control conditions and after incubation with the Aβ1-16 alone or in presence of BTX (**b**). Cumulative distributions of the inter-event intervals of mEPSC from the experiment in **a**. Fragment Aβ1-16 significantly decreased mEPSC inter-event intervals, which is reflected in leftward shift of the cumulative distribution compared to control or Aβ + BTX (p < 0.001, Kolmogorov–Smirnov test). Aβ1-16 increases mean frequency (**c**), without effect on amplitude (**d**), rise time (**e**) and decay time (**f**) of mEPSC. Treatment with BgTx counteracts this effect. **g** Examples of mIPSC traces recorded in control neurons and after incubation with the Aβ1-16. **h** Cumulative distributions of the inter-event intervals of mIPSC in control neurons and after application of Aβ1-16. Aβ1-16 fragment has no effect on the mean frequency (**i**) amplitude (**j**), rise (**k**) and decay time (**l**) of mIPSC. Values in brackets in B-F and in H–L are number of analysed cells obtained from three independent preparations. Data are displayed as means (**b**, **h**) or as means ± SEM (**c**–**l**), *p < 0.05 compared to control, *t* test

To get a direct readout of basal synaptic transmission, we recorded AMPA receptor-mediated miniature postsynaptic currents (mEPSC) using whole-cell voltage-clamp recordings in cultured hippocampal neurons (Fig. 2a). Application of Aβ1-16 (200 pM) 1 h prior to recording significantly increased the mEPSC frequency without changing the current amplitude, rise time or decay time (Fig. 2b-f; frequency (Hz): Ctrl 6.48 ± 1.14, Aβ1-16 10.78 ± 1.73;; amplitude (pA): Ctrl: 22.57 ± 1.66; Aβ1-16: 23.81 ± 1.67; rise *t* (ms): Ctrl: 0.81 ± 0.07, Aβ1-16: 0.73 ± 0.04; decay *t* (ms) Ctrl: 2.80 ± 0.19, Aβ1-16: 2.56 ± 0.14). An application of BgTx (50 nM, 30 min before and during Aβ1-16 application) completely abolished the effect of Aβ1-16 on the mEPSC frequency indicating the involvement of α7nAChR (Fig. 2a–f; Aβ1-16 + BgTx: frequency 4.78 ± 0.94 Hz; amplitude 23.73 ± 3.47 pA; rise *t* 0.71 ± 0.04 ms; decay t 2.66 ± 0.12 ms). Next we examined the effect of Aβ1-16 on GABA receptor-mediated miniature inhibitory postsynaptic currents (mIPSCs) (Fig. 2g). None of the analysed parameters differed between treated cells and controls indicating that Aβ1-16 does not affect GABA-ergic transmission (Fig. 2h–l; frequency (Hz): Ctrl 4.23 ± 1.09, Aβ1-16 3.86 ± ; 1.59; amplitude (pA): Ctrl: 26.76 ± 1.22; Aβ1-16: 30.52 ± 2.23; rise t (ms): Ctrl: 1.48 ± 0.11, Aβ1-16: 1.62 ± 0.11; decay *t* (ms) Ctrl: 16.05 ± 0.99, Aβ1-16: 17.54 ± 1.41). These support the notion that Aβ1-16 affect specifically neurotransmission at excitatory synapses and indicate an involvement of α7nAChR in this process.

### α7nAChRs are required for Aβ-mediated increase in recycling of SVs

To further substantiate the requirement of α7nAChRs for Aβ-mediated increase in TRP, we tested the effect of Aβ in neurons with genetic ablation of these receptors. For this purpose, we cultured cortical neurons derived from Chrna7^flox/flox^ mice having exon 4 of Chrna7 gene (encoding α7nAChR) flanked by lox P sites (Fig. S1A). In these neurons, the expression of functional α7nAChR declined upon infection with nuc-EGFP-cre-recombinase (CRE = CHRNA7 KO) expressing lentiviruses. Cells infected with a lentivirus expressing an inactive form of nuc-EGFP-cre-recombinase (ΔCRE = WT, Figs. S1A, Fig. 2a) were used as control. The efficiency of exon excision was confirmed by qPCR (Fig S1).

**Fig. 3:**
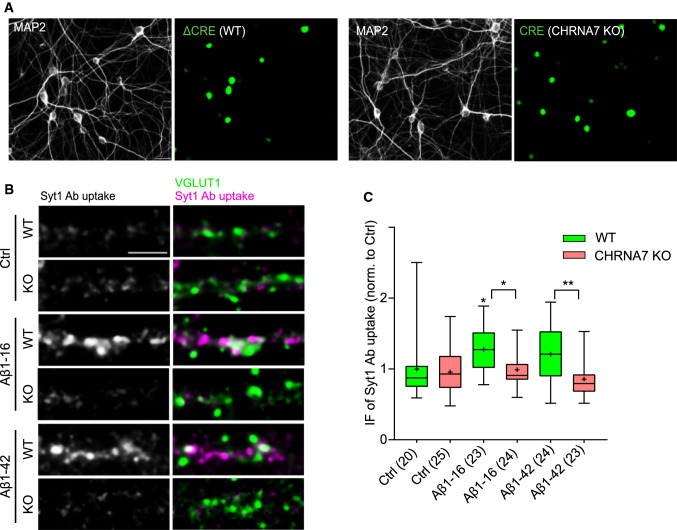
Aβ 1–16-mediated increase in SV recycling requires normal expression of α7nAChRs. **a** Representative images of primary mouse neurons (16 DIV) infected at 4 DIV either with ΔCRE (WT) or CRE (CHRNA7 KO) and stained with MAP2 to assess neuronal transduction efficiency. Scale bar 20 μm. **b** Images show the Syt1 Ab-labelled TRP in cortical mouse cultures (16–17 DIV) pretreated with vehicle (Ctrl), Aβ1-16, or Aβ1-42 on the left (magenta) at excitatory presynapses marked with VGLUT1 (green). Scale bar 5 μm. **c** Quantification of the IF intensity of Syt1 Ab uptake shown in **b**. Values in brackets indicate the number of analysed cells obtained from two independent experiments. Boxes indicate the interquartile range and median, whiskers minimum and maximum values, and + shows the mean. One-way ANOVA followed by Tukey´s post hoc test was used to assess statistical significance. The comparisons to vehicle-treated WT neurons are above each box, the symbols above brackets indicate the effect of CHRNA7-depletion, *p < 0.05, **p < 0.01

Both control (WT) and α7nAChRs-depleted cultures (CHRNA7 KO) showed a comparable size of TRP under basal conditions (Fig. 3b,c; WT: 1 ± 0.10, CHRNA7 KO: 0.96 ± 0.06). Treatment with 200 pM Aβ1-42 and Aβ1-16 for 1 h resulted in an increased TRP in WT (Fig. 3b,c; WT/Aβ1-16: 1.27 ± 0.06; WT/Aβ1-42: 1.21 ± 0.08), but had no effect on CHRNA7 KO cultures (Fig. 3b,c; CHRNA7 KO/Aβ1-16: 0.99 ± 0.05; CHRNA7 KO/Aβ1-42: 0.86 ± 0.05). These results confirm that the regulation of TRP by Aβ depends on α7nAChRs.

### Aβ1-16 modulates nicotine-induced Ca^2+^ influx through α7nAChRs

**Fig. 4:**
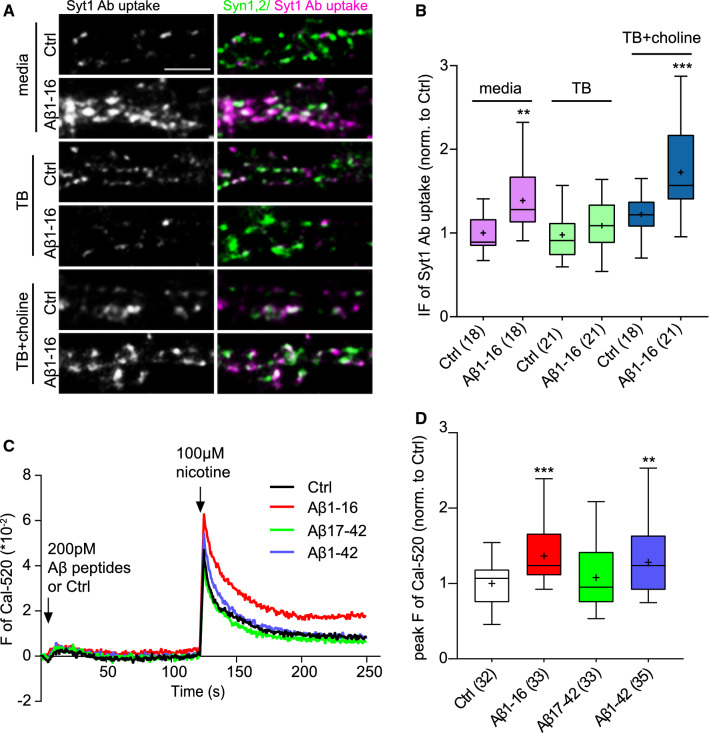
N-terminal portion of Aβ requires choline and nicotine to increase TRP and Ca^2+^ influx via α7nAChRs. **a** Images show TRP labelled by Syt1 Ab loading (magenta) in rat cortical neurons treated either with vehicle or 200 pMAβ1-16 (1 h, 37 °C) in the neuronal growth media, in Tyrode´s buffer or in Tyrode´s buffer supplemented with 28 μM choline. Synapses were identified by co-staining for Syn1,2 (green). Scale bar 5 μm. **b** Quantification of the experiment described in **a**. **c** Ca^2+^ imaging was done using Cal-520 AM indicator in HEK293T expressing α7nAChRs. Traces show the time course of the experiment. Arrows depict the sequential applications of human Aβ1-16, human/rat Aβ17-42, human Aβ1-42 (200 pM, 115 μl/s) or vehicle solution and nicotine (100 μM, 115 μl/s). **d** Quantification of nicotine-induced Cal-520 fluorescence in experiment shown in graph (C). Values in brackets refer to the number of analysed cells (**b)** or recorded wells (**d**) obtained from two (**b**) or four **(D)** independent preparations. Boxes indicate the interquartile range with median, whiskers minimum and maximum values, and + shows the mean. Significance was assessed by one-way ANOVA followed by Dunnett´s post hoc test; marks (***p < 0.001, **p < 0.01) above boxes indicate comparisons to the Ctrl

In the course of our experiments, we observed that neurons treated with Aβ1-16 or Aβ1-42 in culture media showed a robust increase of TRP, but cells from the same batch that were washed and transferred to fresh Tyrode’s buffer (TB) before treatment with the Aβ1-16 peptide failed to increase their TRP (Fig. 4a,b; Aβ1-16: 1.39 ± 0.08; Aβ1-16/TB: 1.09 ± 0.06; Ctrl: 1 ± 0.05). Intriguingly, our neuronal growth medium contains physiological concentration of choline (28 μM), a selective endogenous agonist of α7nAChRs [2]. Thus, we decided to assess whether choline is required for Aβ1-16-induced regulation of TRP in our system. The Syt1 Ab uptake was not different in cells pre-incubated for 1 h in TB or in TB supplemented with choline (Fig. 4a,b; TB: 0.98 ± 0.06; TB + choline: 1.22 ± 0.06). The treatment of cells with Aβ1-16 had no effect on TRP in TB, but cells treated with Aβ1-16 in the presence of choline showed a robust increase in TRP (Fig. 4a,b; Aβ1-16/TB + choline: 1.73 ± 0.11). This indicates that Aβ1-16 likely acts as a positive allosteric modulator of α7nAChRs rather than an agonist.

To test this hypothesis and given the high Ca^2+^ permeability of α7nAChRs [5, 42], we performed a fluorescent-based Ca^2+^ imaging assay in HEK293T cells expressing α7nAChRs and TMEM35A (NACHO), an accessory protein necessary for their efficient surface expression [13]. Cells were loaded with the Ca^2+^-sensitive fluorescent dye Cal-520 AM and recorded using a fluorescent microplate reader. Brief application of both Aβ1-16 and Aβ1-42 produced no changes in fluorescence per se, but significantly enhanced the Ca^2+^ influx induced by application of 100 μM nicotine (Fig. 4c,d; Aβ1-16: 1.37 ± 0.06; Aβ1-42: 1.28 ± 0.07; Ctrl: 1 ± 0.05). In contrast, application of Aβ17-42 had no effect alone and also did not affect the response to nicotine injection (Fig. 4c,d; Aβ17-42: 1.08 ± 0.07). Taken together, these results indicated that the N-terminal but not the C-terminal part of Aβ1-42 acts as a potent allosteric modulator of α7nAChRs enhancing nicotine-induced Ca^2+^ influx.

### Aβ1-16 modulates nicotine-induced inward current of α7nAChRs

**Fig. 5:**
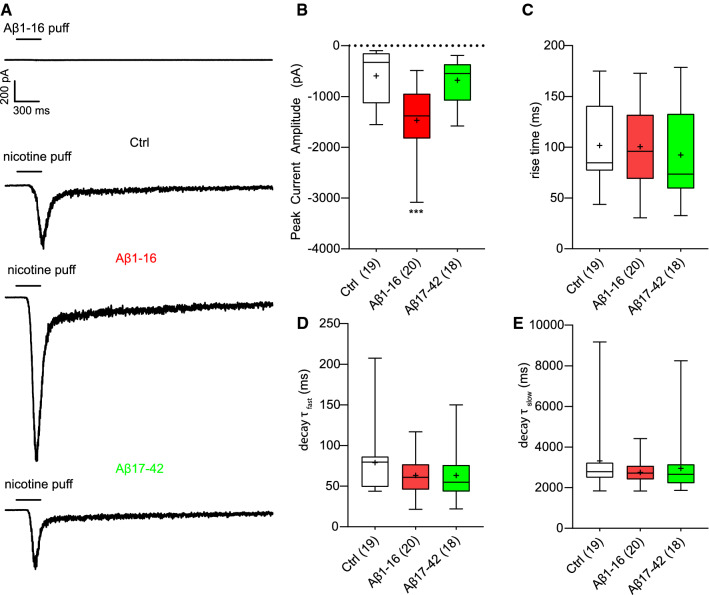
Aβ1-16, but not Aβ17-42, modulates nicotine-induced inward current of α7nAChRs. **a** Representative whole-cell current traces of α7nAChRs transiently expressed in HEK293T cells evoked by 300 ms puffs of 200 pM human Aβ1-16 or by 100 μM nicotine in the presence of vehicle (Ctrl), 200 pM human Aβ1-16 or human/rat Aβ17-42. Cells were held at -70 mV. Quantification of nicotine-evoked peak current amplitude (**b**) and rise time (**c**). Time constant (*τ*) of the fast (**d**) and slow (**e**) component of current decay in response to nicotine puff application in all conditions. *τ* was estimated by fitting the fast and slow phase of current decay with a bi-exponential function. Values in brackets refer to the number of recorded cells. Boxes indicate the interquartile range with median, whiskers minimum and maximum values, and + shows the mean. One-way ANOVA with Dunnett´s multiple comparisons test (**c**) or Kruskal–Wallis test with Dunn’s post hoc test (**b**, **d**, **e**) were used for comparisons to Ctrl; ***p < 0.001.

The modulation of α7nAChRs by N-terminal fragment of Aβ was further examined by whole-cell patch-clamp recordings of nicotine-induced α7nAChRs-mediated currents in HEK293T cells. In this experimental setup, a brief puff application of nicotine reproducibly induced inward currents, while application of Aβ1-16 (200 pM, 300 ms, 2 bar) had no effect (Fig. 5a).

However, if nicotine was applied in presence of Aβ1-16 the evoked inward currents had a significantly higher peak current amplitude (Fig. 5a,b; Aβ1-16: − 1471 ± 156 pA; Ctrl: -594 ± 116 pA). Conversely, the current peak amplitude evoked by nicotine remained unchanged in presence of the C-terminal Aβ17-42 peptide confirming no modulation of α7nAChRs by the C-terminal fragment of Aβ (Fig. 5a,b; Aβ17-42: -680 ± 100 pA). The kinetics of nicotine-induced currents did not significantly differ between treatments. Under all conditions, currents reached their peak within about 100 ms (Fig. 5a,c; Ctrl: 102 ± 8 ms; Aβ1-16: 101 ± 9 ms; Aβ17-42: 92 ± 10 ms) followed by exponentially current decay with a similar fast and slow time constant (*τ*) (Fig. 5a,d,e; *τ*_fast_: Ctrl: 79 ± 9 ms; Aβ1-16: 63 ± 5 ms; Aβ17-42: 63 ± 47 ms; *τ*_slow_: Ctrl: 3320 ± 410 ms; Aβ1-16: 2750 ± 120 ms; Aβ17-42: 2950 ± 330 ms), indicating that Aβ1-16 did not affect channel kinetics. Thus, the N- and not the C-terminal domain of Aβ1-42 acts as a positive allosteric modulator type I of α7nAChRs increasing peak amplitude of nicotine evoked currents but not its desensitization.

### Aβ-derived fragments differently affect phosphorylation of synapsin 1 at serine 551

**Fig. 6:**
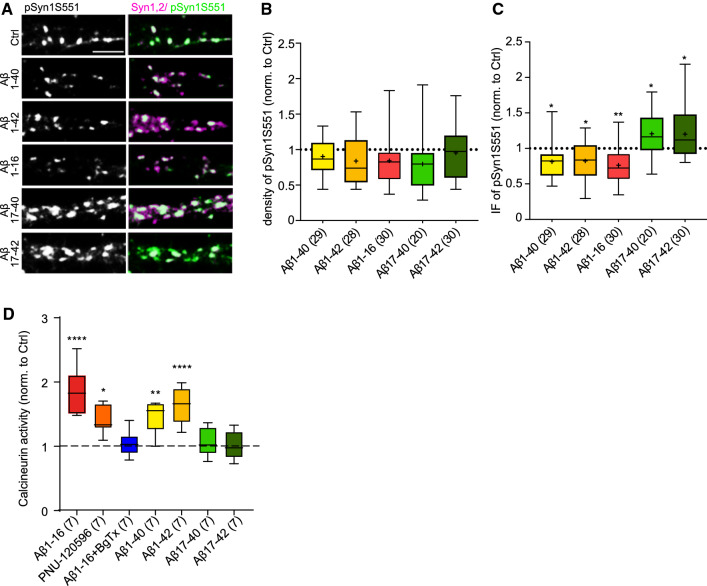
Aβ-derived fragments differently affect phosphorylation of Syn1 at serine 551 and calcineurin activity. **a** Representative images of mature cortical neurons (17–22 DIV) treated with vehicle (Ctrl), rat Aβ1-40, rat Aβ1-42, human Aβ1-16, human/rat Aβ17-40, human/rat Aβ17-42 for 1 h at 37 °C and stained with antibodies recognizing Syn1 phosphorylation at serine 551 (pSyn1S551, green). Syn1,2 (magenta) was used as a synaptic marker. Scale bar 5 μm. **b** Quantification of the density and **c** IF intensity of synaptic puncta positive for pSyn1S551. (D) Quantification of calcineurin activity in cortical neurons (21DIV) treated with Aβ peptides, BgTx and PNU-120596. Data were normalized to the Ctrl. In plots, the boxes show the interquartile range and median, whiskers minimum and maximum values, and + indicates the mean. Values in brackets are the in **b** and **c** number of analysed cells obtained from three independent experiments and in **d** numbers of independent experiments. One-way ANOVA followed by Dunnett´s post hoc test was applied to assess statistical significance of comparison to Ctrl; *p < 0.05, **p < 0.01, ****p < 0.0001

Our previous data indicated a contribution of cyclin-dependent kinase 5 (CDK5)/calcineurin balance in the Aβ-dependent regulation of SV recycling downstream of α7nAChRs [28]. CDK5-mediated phosphorylation of Syn1 at serine 551 (pSyn1S551) controls the transition of SVs from recycling to resting pool [45]. Thus, we assessed possible regulation of this phospho-specific site by Aβ and its fragments. To this end, mature rat cortical neurons were immunostained with an antibody recognizing pSyn1S551 and with a polyclonal anti-Syn1,2 antibody. We found that the number of synaptic puncta positive for pSyn1S551 was unchanged upon treatment with Aβ-derived peptides (Fig. 6a,b; Aβ1-40: 0.90 ± 0.05; Aβ1-42: 0.84 ± 0.06; Aβ1-16: 0.84 ± 0.06; Aβ17-40: 0.80 ± 0.09, Aβ17-42: 0.95 ± 0.06; Ctrl: 1 ± 0.03). However, the synaptic IF intensities were differentially regulated upon application of peptides. While treatment with the peptides containing the first 16 aa of Aβ, namely Aβ1-40, Aβ1-42 and Aβ1-16, significantly decreased the IF intensity of pSyn1S551 as compared to the untreated control (Fig. 6a,c; Aβ1-40: 0.81 ± 0.04; Aβ1-42: 0.82 ± 0.05; Aβ1-16: 0.76 ± 0.04; Ctrl: 1 ± 0.04), an application of Aβ17-42 and Aβ17-40 had opposite effect (Fig. 6a,c; Aβ17-40: 1.21 ± 0.07, Aβ17-42: 1.20 ± 0.06).

The phosphatase that antagonises the effect of CDK5 on S551 is calcineurin [21]. Calcineurin activation is calcium dependent and thus, a good candidate to mediate the Ca^2+^ signalling downstream of α7nAChR. To assess directly, whether physiological Aβ affects the calcineurin activity, we treated cultured neurons with Aβ1-40 and Aβ1-42 peptides and fragments covering their N-, and C-terminal domain, i.e., Aβ1-16 and Aβ17-40 and Aβ17-42, respectively, and measured calcium-dependent phosphatse activity using a selective calcineurin substrate RII and colorimetric detection in lysates form treated cells. The calcineurin activity was significantly increased in cells treated with all fragments contain the N-terminal portion of Aβ including Aβ1-16, Aβ1-40 and Aβ1-42, while the C-terminal fragments Aβ17-40 and Aβ17-42 had not effect (Fig. 6d; Aβ1-16: 1.89 ± 0.14; Aβ1-40: 1.46 ± 0.10; Aβ1-42: 1.62 ± 0.10; Aβ17-40: 1.05 ± 0.08 and Aβ17-42: 1.01 ± 0.08; all normalised to control). Importantly, a co-application of BgTx with Aβ1-16 completely abolished the activation of calcineurin (Fig. 6d; Aβ1-16 + BgTx: 1.05 ± 0.08). An application of PNU-120596 (3 µM), which is well-known positive allosteric modulator type II of α7nAChRs, led to increased calcineurin activity further supporting the key role of α7nAChRs in Aβ1-16-induced calcineurin activation (Fig. 6d; PNU-120596: 1.40 ± 0.08).

Altogether, this data showed a distinct effect of Aβ isoforms on the cellular signalling. Interestingly, the fragments containing the N-terminal part had a distinct effect compared to the peptides covering the C-terminal part. The 16 N-terminal aa were required for calcineurin activation and pSyn1S551 dephosphorylation downstream of α7nAChRs.

### Aβ1-16 increases size of recycling pool to sustain NT release

**Fig. 7:**
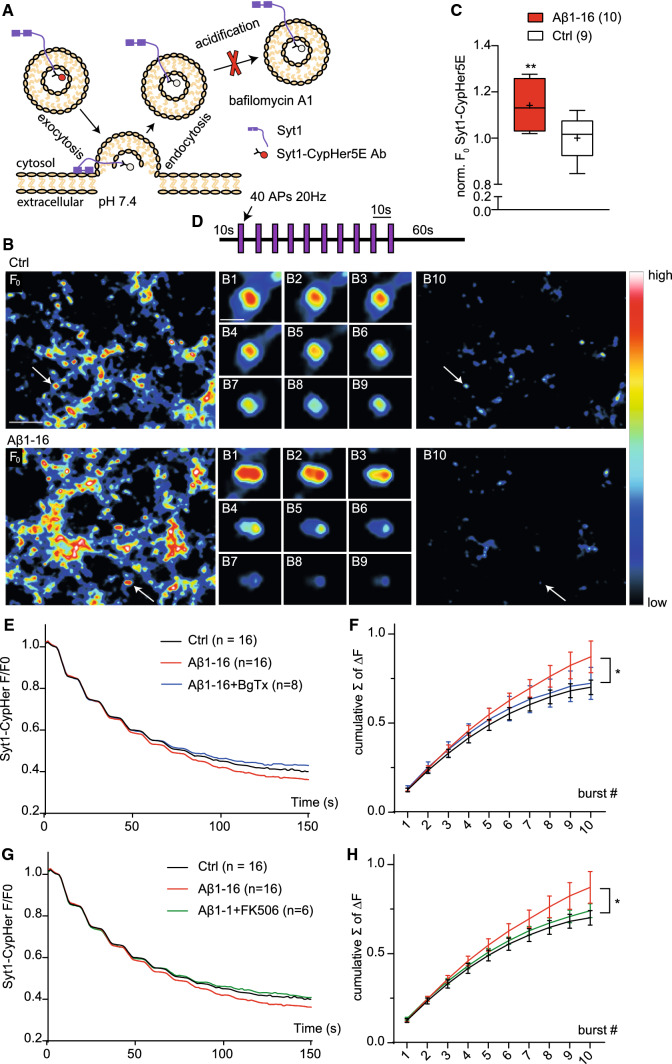
The N-terminal Aβ increases availability of SVs during repetitive stimulation dependently on α7nAChR and calcineurin activation. **a** Schematic representation of the Syt1-CypHer5E imaging. At rest, the fluorescence (F) of CypHer5E is completely unquenched in the low intra-vesicular pH (~ 5.5) and gets quenched upon SV exocytosis in the neutral extracellular media (~ 7.4). Following endocytosis and vesicular reacidification, the CypHer5E F increases again. Blockade of the v-ATPase with bafilomycin A1 prevents vesicular reacidification trapping SVs in the alkaline state. **b** Representative image F_0_ shows typical Syt1-CypHer5E loading in hippocampal neurons (17–20 DIV) incubated with either vehicle (Ctrl) or human Aβ1-16 (200 pM) for 1 h at 37 °C in Tyrode´s buffer with choline before Syt1-CypHer5E loading (1 h, 37 °C). **c** Quantification of the Syt1-CypHer5E loading (*F*_0_). Images **B1–B9** show the course of destaining of an individual synapse (marked by arrow in the F_0_ overview image) upon application of stimulation protocol shown in **d** in the presence of bafilomycin A (1 μM). **B10** shows the whole region upon application of the final stimulus. Scale bar is 5 μm in overview and 1 μm in close-up image. **d** Schematic representation of applied stimulation protocol. After 10 s of baseline, 10 bursts (**B1–B10**) of 40AP at 20 Hz spaced by 10 s pause were applied via electrical field stimulations. Imaging was continued for additional 60 s. **e**,**g** Traces showing average time-course of Syt1-CypHer5E F in response to the stimulation protocol in control and cells treated with Aβ1-16 alone or in presence of either BgTx (E) or FK506 (G). All values were normalized to F_0._ **f**, **h** Cumulative amplitude of Syt1-CypHer5E F calculated from curves shown in **e** and **g**. Data are obtained from at least four independent experiments and are expressed as a mean (**e**, **g**) or mean ± SEM (**f**, **h**) or as boxes depicting the interquartile range and median, with whiskers showing minimum and maximum values, and mean showed as + (**c**). Values in brackets show the number of analysed coverslips from four independent experiments. Unpaired *t* test was used to estimate statistical significance; *p < 0.05, **p < 0.01

To directly address, whether the increased TRP revealed in Aβ-treated neurons by a chemical depolarizing stimulus (4 min, 52.5 mM KCl) contributes to the SV release evoked by electrical activity, we decided to perform live imaging experiments. To this end, we labelled recycling SVs in neurons using Syt1 Ab coupled with a pH-sensitive dye CypHer5E (Syt1-CypHer5E). As depicted in Fig. 7a, this dye exhibits maximal fluorescence in the acidic intravesicular environment of a SV, is quenched upon exocytosis in the neutral pH of extracellular media and becomes visible again following endocytosis and vesicular re-acidification [19]. The neurons treated with Aβ1-16 (in 28 µM choline-containing TB) for 1 h before and during Syt1-CypHer5E showed significantly higher loading (i.e., fluorescence at baseline, F0) than the control (Fig. 7b, c; Aβ1-16: 1.14 ± 0.03; Ctrl: 1 ± 0.03) indicating that a higher number of SVs underwent exo-endocytosis during the labelling period. To analyse the recruitment of SV for neurotransmission during repetitive stimulations, we applied ten consecutive bursts (40 AP-inducing pulses at 20 Hz) spaced by 10 s intervals via field electrodes (Fig. 7d). The applied bursts were shown to release the entire readily releasable pool (RRP) in this neuronal preparation [4]. We performed this experiment in the presence of bafilomycin A1 that blocks vesicular reacidification to visualize release of all newly recruited SVs only (Fig. 7A). Under these conditions, the fluorescence reaches a maximum corresponding the release and alkalization of all SVs that are able to undergo exocytosis during the applied stimulation (Fig. 7d). The decline of fluorescence was more pronounced in Aβ-treated cells (Fig. 7b images B1-B10 and Fig. 7e). The relative change in the fluorescence was calculated for each burst as $\Delta Fx=(Fx\mathrm{max}-Fx\mathrm{min}) / Fx\mathrm{max}$, where *F*_*x*max_ and *F*_*x*min_ express the maximal and minimal fluorescence detected at burst *x*. The cumulative plot of burst-induced changes in fluorescence indicates that a larger proportion of labelled SVs can be mobilized during repetitive stimulations in cells treated with Aβ1-16 compared to control cells (Fig. 7f). Blockade of α7nAChR with BgTx or application of calcineurin inhibitor FK506 completely abolished effect of Aβ1-16 (Fig. 7e–h). Collectively, these data indicate that Aβ1-16 increases availability of SVs for neurotransmission via promoting their recruitment and mobilization for exocytosis and reveal a role of calcineurin signalling downstream of α7nAChR in this process.

## Discussion

Over the past decade, the view on the role of Aβ in the regulation of neuronal function has dramatically changed. Initially, Aβ was considered as a metabolic garbage product which, if not removed properly, forms neurotoxic amyloid plaques that literally clog the brain causing neurodegeneration and cognitive decline. More recently, multiple studies confirmed a beneficial physiological role of Aβ in the regulation of synaptic plasticity and memory [11, 33, 38, 39]. A positive regulatory effect of Aβ in physiological (≈ 200 pM) concentrations on SV recycling has been documented by several previous studies [1, 10, 28]. In the presented study, we set up to investigate the mechanism, by which Aβ exerts this regulation. We dissected the differential effects of N- and C-terminal fragments of Aβ1-40/42. At pM concentrations, the N-terminal fragment Aβ1-16, but not the C-terminal Aβ17-40/42, possessed all positive regulatory properties of high mass Aβ1-40/42 peptides. At the level of individual glutamatergic synapses, it increased the availability of SVs for release by their mobilization and recruitment to the recycling pool. This presynaptic effect was driven by enhancement of calcineurin activity downstream of α7nAChRs leading to dephosphorylation of SV-associated protein Syn1 and consequent reorganization of functional SV pools.

### The N-terminal domain (Aβ1-16) mediates the presynaptic effect of Aβ1-42

In this study, we demonstrate differential effects of N- and C-terminal fragments of Aβ1-42 on regulation of SV recycling. Whereas Aβ1-16, similarly as we have shown previously for Aβ1-42 [28], increased the size of TRP of SVs, the C-terminal fragment Aβ17-42 had no effect. It has been well established that physiological (pM) Aβ1-42 acts as an important endogenous regulator of neurotransmission. Its effects on neuroplasticity were demonstrated in several experimental settings including animal behavioural, electrophysiological recordings of hippocampal LTP as well as optical and electrophysiological studies of synaptic potentiation in cultured neurons and hippocampal slices [1, 14, 28, 38, 39]. Two recent studies indicate the importance of the N-terminal fragment of Aβ1-42 in this process. Richter and colleagues demonstrated that expression of APPsα, but not the APPsβ, increased synaptic plasticity and rescued memory defects in conditional APP/APLP2 KO mice (2018). APPsα and APPsβ are the soluble ectodomains of APP that arise upon its cleavage with α- and β-secretases, respectively, and differ only in the 16 aa sequence covering the Aβ1-16 peptide. A direct application of Aβ1-15 or Aβ1-16 was also able to increase hippocampal LTP in vitro and memory formation in vivo, confirming that the N-terminal domain of Aβ1-42 mediates these neuroplasticity effects [27, 40]. Thus, we propose that the well-documented positive effect of Aβ1-42 on synaptic plasticity and memory resides in its N-terminal domain and most likely relies on the effect of Aβ1-16 on SV recycling. The N-terminal fragments of Aβ ending around the aa 16 are among the most abundant Aβ isoforms found in human CSF [35, 37]. The generation of these N-terminal Aβ isoforms depends on concerted action of α- and β-secretases, but does not involve the γ-secretase [36]. This opens a possibility that activation of a specific APP processing pathway leads to specific regulations of neurotransmission.

### α7nAChRs is the molecular target transducing effects of physiological Aβ1-16

A growing body of evidence supports the role of α7nAChRs in the physiological effect of Aβ1-42 on neurotransmission and neuroplasticity. Probably most convincing are the experiments showing that the enhancement of LTP and memory induced by application of pM Aβ1-42 were completely blocked by a selective antagonist of α7nAChRs BgTx and absent in α7nAChRs KO mice [39]. Our previous study proposed a crucial role for α7nAChRs in the Aβ1-42-dependent bidirectional hormetic regulation of SV recycling in cortical neurons [28]. Here, we confirmed and extended these findings. A pharmacological blockade of α7nAChRs with BgTx or their genetic removal (CHRNA7 KO) completely prevented the increase of TRP of SV at glutamatergic synapses upon application of Aβ1-16 or Aβ1-42.

While a direct binding of Aβ1-42 to α7nAChRs has been indicated by multiple studies [6, 47, 48], the mode of regulation of α7nAChRs by Aβ is a subject of an ongoing debate. While few studies suggest that Aβ acts in a concentration-dependent manner as a direct agonist or antagonist of α7nAChRs [26, 34] our data could not confirm this view. In our hands, Aβ1-16 required physiological concentrations of choline to increase SV recycling in neurons. Moreover, Aβ1-16 and Aβ1-42 failed to induce signalling through α7nAChRs if applied alone, but significantly potentiated nicotine-induced Ca^2+^ influx and inward currents as assessed by Ca^2+^ imaging and whole-cell patch-clamp recordings in HEK293T cells. Thus, we suggest that physiological Aβ1-16 has a modulatory effect on agonist-induced signalling via α7nAChRs. An enhancement of agonist-evoked peak current amplitude of α7nAChRs without changing their gating kinetics indicates that Aβ1-16 acts as a type I positive allosteric modulator of α7nAChRs [12]. This is in line with a previous study showing a significant leftward shift in the nicotine dose–response curve of α7nAChRs upon application of sAPPα, but not of sAPPβ differing only in the Aβ1-16 sequence on their C-terminus [40]. Finally, direct measurement of calcineurin activity in primary neurons revealed that Aβ fragments containing the 16 N-terminal aa elevated activity of this phosphatase. This effect was dependent on activity of α7nAChRs; it was completely blocked by α7nAChR antagonist BTX. In line with this, an application of positive allosteric modulator PNU-120596 had similar affect as Aβ1-16.

Thus, considering the crucial role of Ca^2+^ signalling in the positive effect of Aβ on SV recycling shown previously [28] and in this study, we propose that Aβ controls SV recycling at glutamatergic synapses by modulation of calcineurin activity downstream of cholinergic activation of presynaptic α7nAChRs.

### Aβ1-16 increases TRP via changes in the phosphorylation of SV-associated protein synapsin

Previous work linked the increase of SV recycling upon modulation of physiological Aβ to a dynamic regulation of CDK5/calcineurin balance [28]. CDK5 is well established to decrease size of recycling pool by stabilizing SVs in the resting pool, an effect that is counterbalanced by the Ca^2+^-dependent phosphatase calcineurin [24, 31]. The phosphorylation of serine 551 of Syn1 (pSyn1S551) is dynamically regulated by CDK5 and calcineurin upon Ca^2+^ entry [21]. Verstegen and collaborators showed that the phosphorylation of Syn1 at serine 551 (pSyn1S551) is the molecular target underlying CDK5 effects, since it favours the binding of SV-associated protein Syn1 and actin filaments promoting clustering of SVs into the resting pool (2014). In this work, we extended our previous finding and showed that application of 200 pM Aβ fragments that contain 16 N-terminal aa increased activity of calcineurin downstream of α7nAChRs. Moreover, our data revealed consistent downregulation of pSyn1S551 phosphorylation upon treatment with physiological (200 pM) Aβ peptides containing the N-terminal sequence (Aβ1-16, Aβ1-40, Aβ1-42), which is in line with the observed increase in TRP likely due to a shift of SVs from the resting into recycling pool. Interestingly, the Aβ fragments containing the C-terminal sequence (Aβ17-40, Aβ17-42) increased the pSyn1S551. This might be due to an interference with endogenous Aβ signalling. Finally, the increased SV mobilisation observed upon repetitive stimulation in neurons pre-treated with Aβ1-16 was blocked by co-application of α7nAChR antagonist BgTx or by pharmacological inhibition of calcineurin activity further supporting the significance of calcineurin activation downstream of α7nAChRs in the regulation of SV recycling by physiological Aβ1-16.

Altogether, our data demonstrate an important role for the N-terminal Aβ fragment as a potent enhancer of cholinergic signalling via α7nAChRs. The Ca^2+^ influx through these receptors, activation of phosphatase calcineurin and subsequent change in phosphorylation of Syn1 induces mobilisation of SVs from the resting to the recycling pool, which supports sustained NT release. In AD, high Aβ levels were reported in early stages followed by decreased free extracellular Aβ levels. Both, low or excessive production of the N-terminal Aβ fragment will decrease the dynamic range of signalling via α7nAChRs and thus, lead to deficits in cholinergic modulation. Cholinergic dysfunction is well established in AD. The current pharmacological treatments that, at least intermediately, ameliorate the cognitive decline target cholinergic signalling [17]. Our findings provide a plausible mechanistic explanation for this fact. Moreover, they support the emerging role of physiologically occurring Aβ fragments in cholinergic modulation of glutamatergic neurotransmission.

## Materials and methods

### Animals

Primary rat neuronal cultures used in this study were prepared from E18 embryos of a pregnant Sprague–Dawley rat (RjHan:SD, Janvier Labs, Le Genest Saint-Isle, France), while primary mouse neuronal cultures from new-born homozygous Chrna7^flox/flox^ mice obtained from Jackson laboratories (B6 (Cg)-Chrna7^tm1.1Eh^, #026965*,* Jackson Laboratory, Bar Harbor, Maine, USA) and bred on C57BL/6 J background. All experiments were performed following the European Directive 2010763/EU and according to the local announcement and reporting regulations.

### Antibodies

For immunocytochemistry and live staining, the following antibodies were used: rabbit primary antibodies against synaptotagmin 1 for uptake in mouse cells (Oyster550-labelled, 1:100, #105103C3, Synaptic Systems, Göttingen, Germany), synapsin 1,2 (1:1000, #106002, Synaptic Systems), phospho-synapsin 1 (pSyn1S553) (human pS553 corresponds to pS551 in rat; 1:1000, #ab32532, Abcam, Cambridge, UK), VGLUT1 (rat cultures, 1:1000, #135303, Synaptic Systems), VGAT (1:1000, #131003, Synaptic Systems). Antibodies against synapsin 1,2 (1:1000, #106004, Synaptic Systems) were raised in guinea pig. Antibodies against synaptotagmin 1 for monitoring SV recycling in rat neuronal cells (Oyster550-labelled, 1:250, #105311C3, Synaptic Systems and CypHer5E-labelled, 1:200, #105311CpH, Synaptic Systems), against MAP2 (1:1000, #M4403, Sigma-Aldrich, St. Louis, Missouri, USA) and against VGLUT1 (mouse cultures, 1:250 #135311, Synaptic Systems) were raised in mice. Fluorescent secondary antibodies anti-rabbit Alexa 488 (1:1000, #711545152), anti-guinea pig Alexa 488 (1:1000, #706545148), anti-guinea pig Cy3 (1:1000, #706165148), anti-rabbit Cy3 (1:1000 for rat and 1:500 for mouse cultures #711,165,152) and anti-mouse Cy5 (1:1000 #715175150) were all raised in donkey and purchased from Jackson ImmunoResearch Laboratories (West Grove, Pennsylvania, USA).

### Chemical reagents

Choline chloride (#C7527), nicotine (#N0267) and FK-506 monohydrate (#F4679) were purchased from Sigma-Aldrich. α-Bungarotoxin (BgTx, #11032794) and Bafilomycin A1 (#88899552) from Calbiochem (San Diego, California, USA) and Bicuculline methiodide (Bicu, #120108) from Abcam. d-(−)-2-Amino-5-phosphonopentanoic acid (APV, #0106), 6-Cyano-7-nitroquinoxaline-2,3-dione disodium (CNQX, #0190), Tetrodotoxin (TTX, #1078), PNU-120596 (#2498) and rat Aβ1-40 (#2424, DAEFGHDSGFEVRHQKLVFFAEDVGSNKGAIIGLMVGGVV), rat Aβ1-42 (#2425, DAEFGHDSGFEVRHQKLVFFAEDVGSNKGAIIGLMVGGVVIA) and human Aβ1-42 (#1428, DAEFRHDSGYEVHHQKLVFFAEDVGSNKGAIIGLMVGGVVIA) peptides, were obtained from Tocris Bioscience (Bristol, UK). Human Aβ1-16 (#SPBA16, DAEFRHDSGYEVHHQK), human/rat Aβ17-40 (#SP5043; LVFFAEDVGSNKGAIIGLMVGGVV) and human/rat Aβ17-42 (#SP5044; LVFFAEDVGSNKGAIIGLMVGGVVIA) peptides were purchased from Innovagen (Lund, Sweden). Human Aβ1-42 was used for Ca^2+^ imaging and rat Aβ1-42 for all other experiments. Pilot experiments (data not shown) confirmed that peptides containing the human and rodent N-terminal sequence have indistinguishable effects on SV recycling. All peptides were dissolved in H_2_0 according to the manufacturer´s instructions and stored in small aliquots at − 80 °C. For each experiment, fresh aliquots of peptides were used. H_2_0 was used as vehicle treatment.

### Primary rat neuronal cultures

Primary rat neuronal cultures were prepared as previously described [29]. Pregnant mice were deeply anesthetized with isoflurane (CP-Pharma, Burgdorf, Germany) and sacrificed by decapitation, the E18 embryos were removed by a cesarean section. The brains were collected on ice-cold HBSS−/− (#14175053, Thermo Fisher Scientific, Waltham, Massachusetts, USA) freed from skull and meninges and dissected into cortex and hippocampal regions under microscope (Stemi DV4, Zeiss, Oberkochen, Germany). After treatment with 0.25% (v:v) of Trypsin (#15400054, Thermo Fisher Scientific) for 20 min at 37 °C followed by mechanical trituration in presence of 0.1 mg/ml DNase I (#11284932001, Roche, Basel, Switzerland)*,* cell suspension was filtered through nylons filters (100 μm) and then plated at the required density in DMEM (#41966029, Thermo Fisher Scientific) containing 10% (v:v) fetal calf serum (FCS, #S0015, Biochrom GmbH, Berlin, Germany), 2 mM l-Glutamine (#25030024) and 1% (v:v) Antibiotic/Antimycotic (#15240062) (both Thermo Fisher Scientific). 1 ml cell suspension with a density of 25,000 cells/ml for electrophysiology, 100,000 cells/ml for immunocytochemistry (ICC) and 120,000 cells/ml for live-imaging experiments was plated on poly-l-lysine (PLL, #P1524, Sigma-Aldrich) coated 18 mm Menzel glass coverslips (#6311342, VWR International, Radnor, USA). For calcineurin activity measurements, 500.000 cells were plated per 1 well in 6-well plate. One hour after plating, the medium was replaced by Neurobasal medium (#12348017) supplemented with 2% (v:v) B27 (#17504044), 0.8 mM l-Glutamine and 1% (v:v) Antibiotic/Antimycotic (all from Thermo Fisher Scientific).

### Immunocytochemistry, synaptotagmin 1 antibody uptake assay and image analysis

ICC and Syt1 Ab uptake assay were performed according to an established protocol [29]. For ICC, cortical neurons were fixed in 4% (w:v) paraformaldehyde (PFA) containing 4% (w:v) glucose in PBS for 3 min at room temperature (RT, 22 ± 1 °C), blocked in PBS containing 10% (V:V) FCS and 0.1% (w:v) glycine and next permeabilized with 0.3% (v:v) TritonX 100 in PBS for 40 min. Cells were incubated with primary antibodies overnight at 4 °C and after three PBS washing steps, with secondary antibodies for 1 h at RT. All antibodies were diluted in PBS containing 3% (v:v) FCS. Following incubation, coverslips were washed again three times with PBS and mounted on slides using Mowiol 4–88 (#0713, Carl Roth, Karlsruhe, Germany) or Fluoroshield mounting media without or with DAPI (#F6182 or #F6057, Sigma-Aldrich). For the TRP labelling, the fluorescently labelled antibody against the lumenal domain of Syt1 was applied to the cells in a high KCl-containing Tyrode´s Buffer (TB, in mM: 69NaCl and 52.5 KCl, 2 CaCl_2_, 2 MgCl_2_, 30 glucose, 25 HEPES, pH 7.4) at RT for 4 min. Coverslips were washed three times with physiological TB (in mM: 119 NaCl, 2.5 KCl, 2 CaCl_2_, 2 MgCl_2_, 30 glucose, 25 HEPES, pH 7.4) to remove unbound antibody, fixed, blocked and permeabilized as described before. For each experiment, two coverslips per condition were treated simultaneously with the same reagents and antibodies. All mounted samples were kept at 4 °C until the day of examination. For mouse neuronal cultures, the IF of Syt1 Ab uptake was amplified using an anti-rabbit Cy3-secondary antibody (1:500). 16-bit images were acquired with a 60X/NA1.2 objective (Plan APO VC Nikon CFI, Nikon Corporation, Tokyo, Japan) using an epi-fluorescence microscope (Nikon Eclipse Ti, Nikon Corporation) equipped with an iXon EM + 885 EMCCD Andor camera (Andor Technology, Belfast, UK) controlled by NIS Elements software (Nikon Corporation) or VisiView software (Visitron System GmbH, Puchheim, Germany). After background subtraction, a 16-μm-long region of a proximal dendrite localized 8 μm away from the soma, was semi-automatically selected and cropped in ImageJ (National Institutes of Health, Bethesda, Maryland, USA). Next, synaptic puncta were semi-automatically identified and colocalized with presynaptic markers VGLUT1, VGAT or Syn1,2. Synaptic puncta mean IF intensities were quantified using Open View software kindly provided by N. Ziv [44].

### Production of lentiviral vectors

The active and inactive nuc-EGFP-cre recombinase (CRE and ΔCRE) were kindly provided by P. Kaeser [22]. Lentiviral vectors were produced in HEK293T cells (ATCC, Manassas, Virginia, USA) as described before [9]. Briefly, cells were transfected with the FUGW-based transfer, psPAX2 packaging and pVSVG pseudotyping vectors [30], in a 2:1:1 molar ratio. After 6–8 h, the existing DMEM supplemented with 10% (v:v) FCS, 2 mM l-Glutamine, 1% (v:v) Antibiotic/Antimycotic was replaced by Neurobasal A medium (#12349015, Thermo Fisher Scientific) supplemented with 1% (v:v) GlutaMAX™ (#35050061, Thermo Fisher Scientific), 1 mM sodium pyruvate (#11360070, Thermo Fisher Scientific), 1% (v:v) Antibiotic/Antimycotic and 2% (v:v) B27. 48 h post-transfection, the cell supernatant containing lentiviral vectors was collected and centrifuged at 570 g for 10 min. The supernatant containing lentiviruses was stored at − 80 °C in small aliquots to prevent freeze–thaw cycles.

### Primary Chrna7^flox/flox^ mice cultures

Primary mouse neuronal cultures were prepared according to an established protocol [8] with some modifications. Briefly, newborn α7nAChR^flox /flox^ mice (P0-P1) brains were collected and freed of meninges. Brains were mechanically dissociated by pipetting in a dissociation solution containing 0.127 U/ml of papain (#LK003176, Worthington Biochemical Corporation, Lakewood, New Jersey, USA), 1 mg/ml dispase II (#04942078001, Roche), 0.1 mg/ml DNase I (#LS002139, Worthington), 12.4 mM MgSO_4_ in HBSS−/−, for 10 min at 37 °C, followed by centrifugation for 5 min at 120 g. Cell suspension was filtered through nylon cell strainers (70 μm), resuspended in Neurobasal A-mix media (Neurobasal A supplemented with 1% (v:v) GlutaMAX™, 1 mM sodium pyruvate, 1% (v:v) Antibiotic/Antimycotic and 2% (v:v) B27), following two additional cycles of mechanical dissociation and centrifugation. Prior to culture preparation, 100 µl of Neurobasal A-mix media with 10% (v:v) FCS was applied on the 18 mm-PLL-coated coverslips, and kept at 37 °C till plating. 200,000 cells (in a volume of 100 μl) were plated per coverslip and 1 ml of fresh Neurobasal A-mix media was added 1 h later. All cells were maintained at 37 °C in a humidified incubator containing 5% CO_2_. Cells were transduced with CRE or ΔCRE encoding lentivirus at 4 days in vitro (DIV).

### qPCR to assess the cre-mediated Chrna7 deletion in Chrna7^flox/flox^ mice cultures

Total RNA was extracted from cultures 14 days after viral transduction using Pure Link RNA mini kit (#12183018A, Life technologies, Carlsbad, California, USA), according to the manufacturer’s instructions. The cDNA was synthetized according to SuperScript VILO cDNA Synthesis kit (#11754050, Invitrogen). qPCR was performed using SYBR-green (#04707516001, Roche) and a Light cycler 480 II (Roche). Primers were designed to amplify the sequence spanning Chrna7 exon 3–5 (primer pair 1 (PP1): For: 5´- TGAGAAGAACCAAGTTTTAACCACC-3´; rev: 5´-ACCAAGACGTTGGTGTGGAA-3´) and exon 4–5 sequence (primer pair 2 (PP2): for: 5´- TTCGTTTTCCAGATGGCCAGA-3´; rev: 5´-ACCAAGACGTTGGTGTGGAA-3´). Glyceraldehyde-3-phosphate dehydrogenase (GAPDH) was used as internal control: (for: 5´-CGACTTCAACAGCAACTCCCACTCTTCC-3´; rev: 5´-TGGGTGGTCCAGGGTTTCTTACTCCTT-3´). The expression level of Chrna7 was normalized to GAPDH and analysed using the delta-delta Ct method using Light Cycler 480 software (version 1.5, Roche). The size of the qPCR products obtained with PP1 was assessed by agarose gel electrophoresis and the optical density was quantified in image studio software (LI-COR Bioscience, Lincoln, Nebraska, USA).

### Culture and transfection of HEK293T cells

HEK293T (ATCC) cells were cultured in DMEM supplemented with 10% (v:v) FCS, 2 mM l-Glutamine and 1% (v:v) of Antibiotic/Antimycotic. At 70% confluence, cells in T25 flask were transfected using jetPEI kit (#101, Polyplus transfection, Illkirch, France) according to manufacturer’s instructions. For Ca^2+^ imaging, HEK293T cells in a T25 flask were co-transfected with 2 μg of each pcDNA3.1-CHRNA7, (#62276, Addgene, Watertown, Massachusetts, USA, [49]), pCMV6-XL5-TMEM35A (#SC112910, OriGene Technologies, Rockville, Maryland, USA) together with 75 ng of pCVM-mCherry-N1 (#632523, Clontech, Mountain View, California, USA) plasmids. For electrophysiological studies, 1 μg of each pcDNA3.1-CHRNA7, pCMV6-XL5-TMEM35A and 75 ng pEGFP-C2 (#6083–1, Clontech, Mountain View, California,USA) were transfected. 48 h post-transfection, cells were plated either on 96-well PLL-coated clear-bottom black well plates (#3603, Corning, New York, USA) at a density of 140,000 cells/well and used for Ca^2+^ imaging experiments on the next day or in 35 mm TC-treated cell culture dishes (#353001, Corning) for electrophysiology, and recorded on the same day.

### Calcium imaging

Transfected HEK293T cells were loaded with 4 μM of Cal-520® AM (#21130, AAT Bioquest, Sunnyvale, California, USA) at 37 °C for 75 min in Ca^2+^ assay buffer (in mM: 137 NaCl, 4 KCl, 2 CaCl_2_, 1 MgCl_2_, 5 glucose, 10 HEPES, 2 probenecid (#P8761, Sigma-Aldrich), pH 7.4) supplemented with 0.2 mg/ml Pluronic® F-127 (#20050, AAT Bioquest). The background fluorescence was determined from unloaded cells. The intracellular Ca^2+^ influx was recorded using the well mode and the direct optic bottom reading (ex: 483–14 em: 530–30) setting on the CLARIOstar microplate reader (BMG Labtech, Ortenberg, Germany). After 3 s of baseline, Aβ1-16, Aβ1-42, Aβ17-42 or vehicle solution were applied using one on-board reagent injector to achieve final concentration of 200 pM. After 2 min of recordings, nicotine was applied using the second on-board reagent injector to final concentration 100 μM and the measurements were taken for other 2 min every 0.5 s.

For analysis, the averaged background fluorescence measured in unloaded cells was subtracted from each data point. The average of the baseline before stimulation (*F*_0_) was subtracted from each raw fluorescence intensity (*F*) and all data were normalized to F_0_, according to the formula: (*F *− *F*_0_)/*F*_0._

### Whole-cell patch clamp

Transfected HEK293T cells were identified with a 40X objective using an inverted microscope (Axiovert 40, Zeiss) equipped with a fiber-optic-coupled light source (UVICO, Rapp OptoElectronic, Hamburg, Germany). Cells were immersed in extracellular solution containing in mM: (142 NaCl, 4 KCl, 2 Mg_2_Cl_2_, 2 CaCl_2_, 10 HEPES, 10 glucose, pH 7.4, 330 mosm/l) and 3 min after whole-cell access was established, α7nAChRs currents were recorded at RT (22 ± 1 °C) in whole-cell voltage-clamp mode using an Axopatch 700B amplifier in conjunction with a Digidata 1322A interface controlled by pClamp 10 software (all from Molecular Devices, San Jose, California, USA). Membrane potential was held at − 70 mV. Borosilicate glass pipettes with filament (BioMedical Instruments, Zöllnitz, Germany) were pulled with a DMZ-Universal puller (Zeitz-Instruments, Planegg, Martinsried, Germany) and filled with internal solution (containing in mM: 135 K-gluconate, 5 HEPES, 4 NaCl, 3 MgCl_2_, 5 EGTA, 2 Na_2_-ATP, 0.3 Na_3_-GTP, pH 7.25, 280 mosm/l). Pipette resistance measured in bath solution was < 5 MΩ before series resistance compensation (75%). Nicotine or Aβ1-16 were applied in extracellular solution at concentration of 100 μM or 200 pM respectively, using a glass pipette with a PDES-02I pneumatic drug ejection system (npi electronic, Tamm, Germany) (300 ms, 2 bar) controlled by the command protocol. The resistance of the puff pipette measured in bath solution was always > 3 and < 5 MΩ. In a different experiment, Aβ1-16 or Aβ17-42 or vehicle solution were applied to the bath solution at a final concentration of 200 pM and α7nAChRs currents were recorded following puff application of nicotine. Signals were digitized at 20 kHz and filtered at 5 kHz. Leak subtraction was performed offline.

Hippocampal neurons used for whole-cell patch clamp recordings were grown for 14–18 days. Patch pipettes from borosilicate glass had a pipette resistance of 4–6 MΩ using Sutter P97 puller (Sutter Instrument, Novato, California, USA). Recordings with the series resistance ≥ 20 MΩ were discarded from the analysis. Extracellular solution contained in mM: 145 NaCl, 2.5 KCl, 2 MgCl2, 2 CaCl2, 10 HEPES, and 10 d-glucose (pH 7.4 adjusted with NaOH). Intracellular solution for recording of miniature EPSC (mEPSCs) contained in mM: 140 K-gluconate, 1 MgCl2, 2 CaCl2, 4 NaATP, 10 EGTA, 10 HEPES, and 2 Mg-ATP, 0.3 Na-GTP (pH 7.2–7.3, adjusted with KOH). Miniature IPSCs were recoded using intracellular solution in mM: 118 KCl, 9 EGTA, 10 HEPES, 4 MgCl2, 1CaCl2, 2 Mg-ATP, 0.3 Na-GTP, (pH 7.2–7.3 adjusted with KOH). For the recording of mEPSCs 1 µM TTX, 25 µM APV, and 10 µM Bicu, were added to the bath solution to block Na-channels, NMDA-receptors and GABAA-receptors respectively. To isolate mIPSCs, 10 µM CNQX were added to the bath solution to block AMPA receptors. The holding potential for mEPSCs and mIPSCs recordings neurons was -60 and -70 mV, respectively. Aβ1-16 (200 pM) were added to culture medium of cells kept at 35 °C 1 h prior to experiment and were also present in the extracellular solution during the recording. BgTx (50 nM) was added to the culture medium 30 min prior to the amyloid peptides. mEPSCs and mIPSCs (200 events per cell) were recorded 60 s after the establishment of whole-cell configuration and analysed using Mini Analysis Program (Synaptosoft, Decatur, Georgia, USA). Data were acquired at 22 °C, using an EPC10 double Patch-clamp amplifier, filtered at 3 kHz and sampled at 40 kHz using Patch Master 2.32 software and analyzed by use of Fit Master software v2.69 (all from HEKA, Lambrecht, Germany). Cultures from at least three independent preparations were used for each condition.

### Calcineurin activity measurements

Calcineurin activity was determined using the fluorimetric calcineurin activity assay kit (#207007, Calbiochem) according to the manufacturer’s instructions and the Clariostar (BMG) plate reader. Briefly, cortical neurons were grown for in six-well plates for 21 days. 1 h before treatments growth medium was changed to Tyrode’s buffer (TB) (in mM: 120 NaCl, 5 KCl, 10 glucose, 18 NaHCO_3_, 1 MgCl_2_, 2.5 CaCl_2_, pH 7.4) supplemented with 28 µM choline. BgTx (50 nM) was added 30 min after the medium change. Aβ peptides (200 pM) and PNU-120596 (3 µM) were added 1 h after medium change and kept for 1 hr at 37 °C at 5% CO2 atmosphere. In total 2 h after medium change, cells in all wells were harvested in 150 μl of kit lysis buffer with protease inhibitor cocktail. For each experiment three wells were pooled. The lysates were processed over Zeba spin column (#89883, ThermoFisher Scientific) to remove free phosphate and exchange buffer to kit assay buffer. To calculate calcineurin specific activity, the Ca2 + -independent phosphatase activity measured in the presence of EGTA was subtracted from the total phosphatase activity measured in lysates. Protein concentration in each lysate was determined using standard BCA assay and the calcineurin activity was corrected for total protein amounts in respective sample. Relative activities were normalized to the untreated control.

### Anti-synaptotagmin 1 Ab-CypHer5E imaging and analysis

To label all recycling vesicles, primary rat hippocampal cultures (18–21 DIV) were incubated with anti Syt1-CypHer5E Ab for 1 h at 37 °C in TB supplemented with 28 µM choline. After loading, cells were washed twice with TB to remove excessive dye. Treatments were done as follows: Aβ1-16 was applied 1 hr prior to and during Syt1-CypHer5E Ab loading in TB supplemented with 28 µM choline. BgTx (50 nM) was applied 30 min before Aβ1-6 and kept throughout the whole experiment. To inhibit calcineurin activity FK 506 (1 µM) was added along with Aβ1-16 treatment. Aβ1-16 and inhibitors were kept in cells also during the imaging. The application of FK506 alone had no effect on Syt-CypHer5E release (not shown). For imaging, coverslips were placed on the base of a field stimulation chamber equipped with 10 mm spacing platinum electrodes (#RC-49MFSH without perfusion, Warner Instrument, Hamden, Connecticut, USA) and imaged at RT in physiological TB supplied with 10 μM CNQX, 50 μM APV, 1 μM bafilomycin A1 and 28 µM choline on epifluorescence microscope (Nikon Eclipse Ti, Nikon Corporation), using an automated perfect focus system (PFS) and 60X/NA1.2 water-immersion objective (CFI Plan Apo VC, Nikon Corporation). To release the readily releasable pool (RRP), 40 APs were evoked by electric field stimulation (1 ms pulses of 70 mA) applied by A 385 stimulus isolator (World Precision Instruments, Sarasota, Florida, USA) connected to a stimulus generator (#STG-4008, Multi Channel Systems, Reutlingen, Germany). Subsequent to stimulations TB containing 60 mM NH_4_Cl was applied to achieve alkalization across all membranes. CypHer5E fluorescent dye was excited at 628/40 nm with a Led-HUB lamp (Omicron-laserage Laserprodukte GmbH, Rodgau, Germany) and time-lapse images were acquired at the frequency of 1 Hz using Andor camera (Andor Technology) controlled by VisiView (Visitron System GmbH) software in 2 * 2 binning mode.

Data were exported as stack files (.stk) containing frames with 512 × 512 pixels of 16-bit monochromatic intensity values. For data processing, files were converted to tagged image file format (Multipage-tiff) using ImageJ (National Institutes of Health) and loaded to MATLAB® to identify active boutons and fluorescence changes upon stimulation by custom-written scripts. A feature point detection algorithm [41] was implemented to automatically detect regions of interest (ROIs). In brief, coordinates of ROIs were identified on the averaged image formed from three frames prior to the first electrical stimulation. First, centroids of ROIs were determined as local fluorescence intensity maxima additionally exceeding an intensity threshold value set by distribution of intensity values. ROIs are represented as pixels circularly surrounding the detected centroids. Fluorescence intensity traces of individual ROIs were obtained by averaging fluorescence intensity from pixels within corresponding ROI in each frame. For background subtraction, background intensity value was determined on each frame by smoothing with a Gaussian filter and thresholding the grey level histogram to select dark pixels. The mean value over dark pixels was subtracted from the corresponding original frame. Dye bleaching was computationally compensated by modelling the effect with an exponential fit of a bleaching curve according to {Hua, 2011 #1}. Therefore, bleaching curve was constructed from averaged ROI derived traces. Within this mean curve, segments displaying a strong change in fluorescence upon stimulation were detected by a custom-written edge detection function returning positions of these segments as well as related change in fluorescence ΔF. Detection is based on the second derivative to determine the edges within the curve. Finally, detected segments were cut out and remaining sections were assembled by adding the cumulative fluorescence difference of related stimulus. An exponential Eq. (1) modelled bleaching related decay in the mean curve. Numerical fit with MATLAB® built-in functions yielded in coefficient lambda λ.

1 $$Y \, = \, a*\exp \left( {\lambda *x} \right)$$

Finally, iterative deconvolution according to formula (2) corrected for bleaching with I_Measure_(i) being the intensity value of the present curve at data point i and I_corr_ being the corrected intensity value at data point.

2 $$I_{{{\text{corr}}}} \left( i \right) \, = \, I_{{{\text{Measure}}}} \left( i \right) \, - \, \Sigma \left( {\lambda * \, I_{{{\text{Measure}}}} \left( i \right)} \right)$$

Fluorescence traces were normalized by min–max feature scaling according to (3). Thereby *I*_min_ is related to the fluorescence intensity after NH_4_Cl alkalization and corresponds to the averaged values from the last three frames of a recording. Accordingly *I*_max_ is the baseline value prior to electrical field stimulations and corresponds to the averaged values derived from frames three to six.

3 $$I^{\prime}= \frac{{I} - {{I}}_{\text{min}}}{{{I}}_{\text{max}}-{{I}}_{\text{min}}}$$

To exclusively evaluate active synaptic boutons that release synaptic vesicles upon electrical field stimulation, traces from individual ROIs were analysed to get precise positions and values of ΔF upon stimulation. Therefore a modified algorithm based on the edge detection algorithm from bleaching correction was used. Invalid positions that were detected due to the high noise within individual traces were sorted out by passing the positions from the previously performed edge detection as template to the program. Finally, ROIs were characterized as active synaptic boutons if first or second ΔF exceeds a threshold that was defined as the median of the 20% lowest ΔF across all ROIs within the whole recording. Additionally, ROIs with less than four responses out of ten stimulations were excluded. Mean curve from active synaptic boutons was calculated and mean ΔF upon each electrical was determined for each stimulation by performing edge detection algorithm again. Results were then exported as Excel file (Microsoft, Redmond, Washington, USA) for statistics and visualization in Excel and GraphPad Prism 8. All used codes are freely available on Github (https://github.com/EvaMWe/CypHer5E Imaging).

### Statistics

Statistical analyses were performed using GraphPad Prism 8 (GraphPad Software, San Diego, California, USA). All data are normalized as described in graphs, expressed as mean ± SEM in text and represented as `Box-whiskers´ showing the interquartile distance with median, minimum and maximal values and mean showed as + in plots. The statistical tests and numbers of cells (ICC, Syt1 Ab uptake assay, and electrophysiology data), number of wells from a 96-well plate (Ca^2+^ data), coverslips (Syt1- CypHer5E data) and independent cultures preparation (qPCR data) analysed for each experiment are indicated in the respective figure legend and graphs. Statistical significances are showed as *p < 0.05, **p < 0.01, ***p < 0.001.

### Supplementary Information

Below is the link to the electronic supplementary material.

Figure S1 Viral CRE expression efficiently diminishes expression of Chrna7 in cultured neurons from Chrna7flox/flox mice. (A) Schematic representation of the Chrna7 floxed allele. LoxP sites flanking the exon 4, primer locations and expected PCR products are shown for intact and recombined gene. Primer pair 1 (PP1) encompassing exon 3-5 yields two fragments of 198 (floxed allele) and 88 bp (recombined allele) in neurons transduced with the CRE-lentivirus and only one fragment of 198 bp in neurons transduced with ΔCRE. Using primer pair 2 (PP2), PCR product (97 bp) was expected only in the foxed allele, but not upon cre-mediated recombination. (B) Agarose gel electrophoresis showing RT-PCR products (run in triplicates) obtained using PP1 from three independent preparations of neurons derived from Chrna7flox/flox mice and infected either with CRE or ΔCRE. The upper and the lower bands correspond to the floxed (198 bp) and recombined allele (88 bp), respectively, obtained from three independent cultures preparation. (C) Quantification of optical density (OD) for the 198 bp fragment, corresponding to the floxed gene, from the gel shown in (B) indicates down-regulation of Chrna7 expression by more than 80% upon CRE infection compared to ΔCRE (CRE: 0.14 ± 0.02; ΔCRE: 1 ± 0.07). (D) Expression of the mRNA levels of the floxed allele measured by qPCR using PP2 on primary mouse neuronal cultures infected either with CRE or ΔCRE. Transduction with CRE decreased expression of floxed Chrna7 by 70% as compared to ΔCRE, demonstrating an efficient Chrna7 deletion in our in vitro system (CRE: 0.26 ± 0.03; ΔCRE: 1 ± 0.16). Values in brackets indicate number of independent preparations. An unpaired t-test was used to assess statistical significance, *p < 0.05, ***p < 0.001 (PDF 138 kb)

## Data Availability

Data and material are available upon reasonable request.

## Code availability

All used codes are available on Github (https://github.com/EvaMWe/CypHer5E Imaging).
