# Supplementary figures and images for: Aβ1-16 controls synaptic vesicle pools at excitatory synapses via cholinergic modulation of synapsin phosphorylation
Source: Cell Mol Life Sci. 2021 Apr 17;78(11):4973–92. doi: 10.1007/s00018-021-03835-5 (PMC8233295; doi:10.1007/s00018-021-03835-5)

**Figure S1**

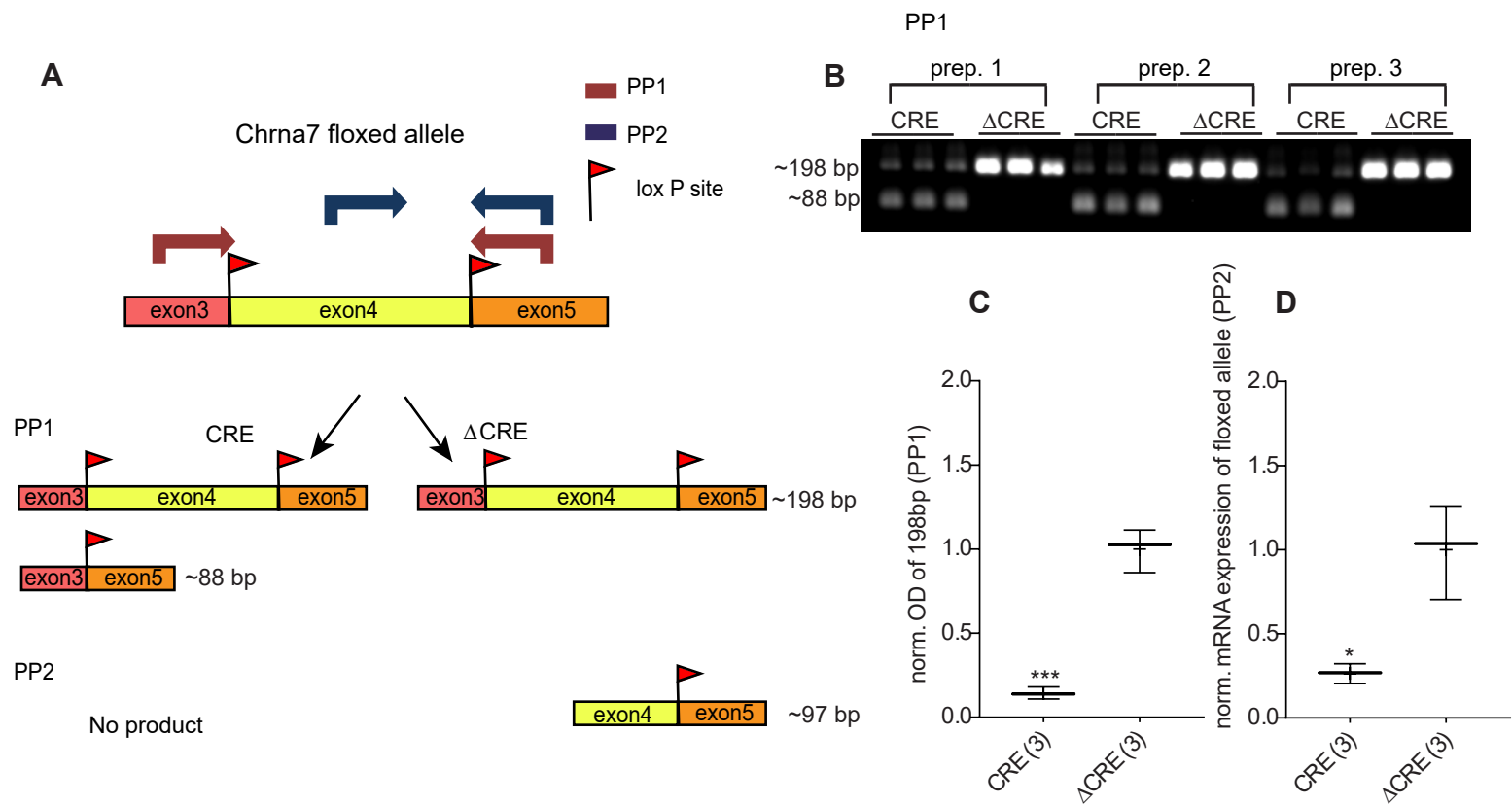

Supplement: Supplementary file 1 — Figure S1 Viral CRE expression efficiently diminishes expression of Chrna7 in cultured neurons from Chrna7flox/flox mice. (A) Schematic representation of the Chrna7 floxed allele. LoxP sites flanking the exon 4, primer locations and expected PCR products are shown for intact and recombined gene. Primer pair 1 (PP1) encompassing exon 3-5 yields two fragments of 198 (floxed allele) and 88 bp (recombined allele) in neurons transduced with the CRE-lentivirus and only one fragment of 198 bp in neurons transduced with ΔCRE. Using primer pair 2 (PP2), PCR product (97 bp) was expected only in the foxed allele, but not upon cre-mediated recombination. (B) Agarose gel electrophoresis showing RT-PCR products (run in triplicates) obtained using PP1 from three independent preparations of neurons derived from Chrna7flox/flox mice and infected either with CRE or ΔCRE. The upper and the lower bands correspond to the floxed (198 bp) and recombined allele (88 bp), respectively, obtained from three independent cultures preparation. (C) Quantification of optical density (OD) for the 198 bp fragment, corresponding to the floxed gene, from the gel shown in (B) indicates down-regulation of Chrna7 expression by more than 80% upon CRE infection compared to ΔCRE (CRE: 0.14 ± 0.02; ΔCRE: 1 ± 0.07). (D) Expression of the mRNA levels of the floxed allele measured by qPCR using PP2 on primary mouse neuronal cultures infected either with CRE or ΔCRE. Transduction with CRE decreased expression of floxed Chrna7 by 70% as compared to ΔCRE, demonstrating an efficient Chrna7 deletion in our in vitro system (CRE: 0.26 ± 0.03; ΔCRE: 1 ± 0.16). Values in brackets indicate number of independent preparations. An unpaired t-test was used to assess statistical significance, *p < 0.05, ***p < 0.001 (PDF 138 kb) [file 18_2021_3835_MOESM1_ESM.pdf]
